# Deficiency of Urokinase Plasminogen Activator May Impair β Cells Regeneration and Insulin Secretion in Type 2 Diabetes Mellitus

**DOI:** 10.3390/molecules24234208

**Published:** 2019-11-20

**Authors:** Chung-Ze Wu, Shih-Hsiang Ou, Li-Chien Chang, Yuh-Feng Lin, Dee Pei, Jin-Shuen Chen

**Affiliations:** 1 Division of Endocrinology and Metabolism, Department of Internal Medicine, School of Medicine, College of Medicine, Taipei Medical University, Taipei 11031, Taiwan; chungze@yahoo.com.tw; 2 Division of Endocrinology and Metabolism, Department of Internal Medicine, Shuang Ho Hospital, Taipei Medical University, New Taipei City 23561, Taiwan; 3 Division of Nephrology, Department of Internal Medicine, Kaohsiung Veterans General Hospital, Kaohsiung 81362, Taiwan; blueyeou1104@gmail.com; 4 School of Pharmacy, National Defense Medical Center, Taipei 11490, Taiwan; lichien@ndmctsgh.edu.tw; 5 Graduate Institute of Clinical Medicine, College of Medicine, Taipei Medical University, Taipei 11031, Taiwan; linyf@ndmctsgh.edu.tw; 6 Deputy Superintendent, Shuang Ho Hospital, Taipei Medical University, New Taipei City 23561, Taiwan; 7 Division of Endocrinology and Metabolism, Department of Internal Medicine, Fu Jen Catholic University Hospital, New Taipei City 24352, Taiwan; peidee@gmail.com; 8 School of Medicine, College of Medicine, Fu Jen Catholic University, New Taipei City 24205, Taiwan; 9 Department of Education and Research, Kaohsiung Veterans General Hospital, Kaohsiung 81362, Taiwan

**Keywords:** urokinase plasminogen activator, type 2 diabetes mellitus, insulin secretion, β cell regeneration

## Abstract

Background: The relationship between urokinase-type plasminogen activator (uPA) and the development of type 2 diabetes mellitus (T2DM) was investigated in the study by using mice and cell models, as well as patients with T2DM. Methods: In mice models, wild-type and uPA knockout (uPA-/-) BALB/c mice were used for induction of T2DM. In cell models, insulin secretion rate and β cell proliferation were assessed in normal and high glucose after treating uPA siRNA, uPA, or anti-uPA antibody. In our clinical study, patients with T2DM received an oral glucose-tolerance test, and the relationship between uPA and insulin secretion was assessed. Results: Insulin particles and insulin secretion were mildly restored one month after induction in wild-type mice, but not in uPA-/- mice. In cell models, insulin secretion rate and cell proliferation declined in high glucose after uPA silencing either by siRNA or by anti-uPA antibody. After treatment with uPA, β cell proliferation increased in normal glucose. In clinical study, patients with T2DM and higher uPA levels had better ability of insulin secretion than those with lower uPA levels. Conclusion: uPA may play a substantial role in insulin secretion, β cell regeneration, and progressive development of T2DM. Supplementation of uPA might be a novel approach for prevention and treatment of T2DM in the future.

## 1. Introduction

In recent decades, cases of type 2 diabetes mellitus (T2DM) have been rapidly increasing worldwide. While the causes of T2DM include insulin resistance and impaired insulin secretion, some clinical evidence has shown progressive insulin secretion impairment over time in patients with T2DM [1,2,3]. The causality of mass declination of β cells in T2DM is the increasing rate of apoptosis rather than neogenesis [4]. However, the factors associated with apoptosis and neogenesis in β cells are still unknown. Preventing the development of T2DM and β cell dysfunction is an important issue in controlling T2DM.

Urokinase-type plasminogen activator (uPA) is well-known as a serine protease which activates plasminogen, converting to plasmin in the fibrinolytic process of thrombosis and extracellular matrix. Patients with T2DM are prone to formation of intravascular thrombosis and an imbalance between coagulation and fibrinolysis is implicated in the pathogenesis [5]. In addition to triggering a fibrinolytic cascade, uPA is a pleiotropic functional protein linked to innate immune response in regulating immune cell migration and recruitment [6,7]. The uPA independently enhances release of some inflammatory cytokines and activates matrix metalloproteinase [8]. Moreover, uPA also plays an important role in tissue remodeling and atherosclerosis [9,10].

Clinically, intravenous uPA is commonly used for thrombolytic therapy on peripheral artery occlusion in patients with T2DM. High glucose reduces uPA activity and degradation of extracellular matrix on mesangial cells [11]. In addition, the expression of uPA declines during development of adipose tissue in mice fed a high-fat diet, whose blood glucose is elevated time-dependently [12]. Furthermore, local application of exogenous uPA accelerates wound healing in the diabetic mouse model [13], and islet surface modification with uPA, instead of heparin, improves β cell survival on transplantation of islets [14]. However, except for these weak relationships, the role of uPA on T2DM is unknown.

In this study, we investigated the causal relationship between uPA and T2DM by using wild-type and uPA knockout (uPA-/-) mice, indexing the diabetes-induced rate, insulin resistance, and insulin secretion. We also explored the β cell proliferation and insulin secretion after silencing expression of uPA or treating with uPA, respectively, in in vitro cell models. In the clinical cases, we analyzed the relationship between uPA levels and insulin secretion in patients with T2DM. The aim of this study was to explore the role of uPA in T2DM.

## 2. Results and Discussion

### 2.1. uPA-/- Mice Were Prone to HyperGlycemia and Development of T2DM

**Figure 1:**
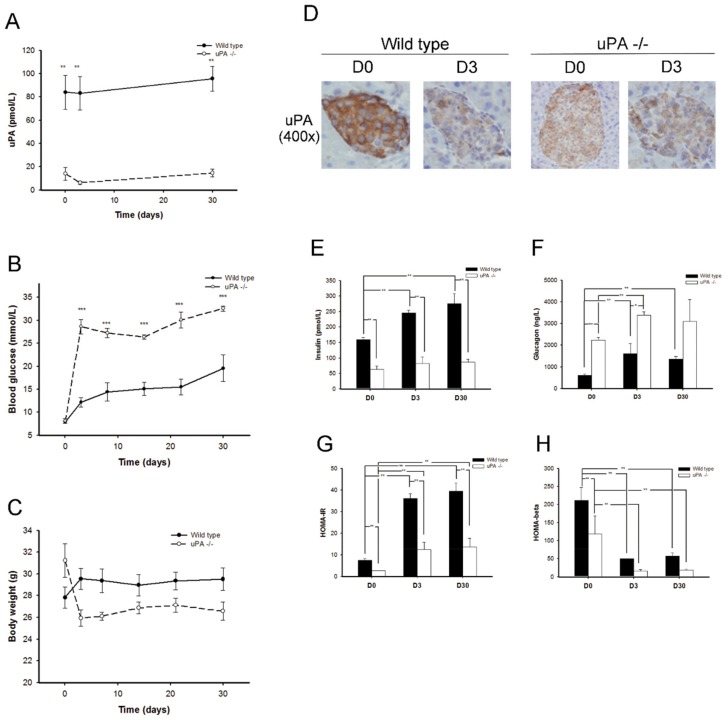
Changes in uPA, blood glucose, body weight, uPA expression on islet, insulin, glucagon, insulin resistance (HOMA-IR), and insulin secretion (HOMA-β) before and after development of type 2 diabetes mellitus (T2DM) in both wild-type and uPA-/- mice (n ≥ 4). (**A**) The uPA levels in wild-type (solid line) mice were significantly higher than those in uPA-/- (dashed line) BALB/c mice. ^**^ *p* < 0.01. (**B**) D0, blood sugar levels in both uPA-/- and wild-type mice were similar. After induction, blood glucose in uPA-/- BALB/c mice was significantly higher than in wild-type mice. ^***^ *p* < 0.001. (**C**) The body weight in both wild-type and uPA-/- BALB/c mice showed no significant difference. (**D**) The expressions of uPA on islet before and after induction of T2DM were compared by immunohistochemical stain. In wild-type mice, uPA expression obviously decreased after induction of T2DM. The low expressions of uPA persisted both before and after induction in uPA-/- mice. (**E**) The insulin levels in wild-type mice were significantly higher than in uPA-/- mice. With the high-fat diet, insulin levels increase progressively in both wild-type and uPA-/- mice. (**F**) The glucagon levels declined gradually, along with elevation of insulin levels in wild-type and uPA-/- mice. (**G**) The HOMA-IR in wild-type mice was significantly higher than in uPA-/- mice. With a high-fat diet, the HOMA-IR increases significantly and progressively in both wild-type and uPA-/- mice. (**H**) The HOMA-β in uPA-/- mice was lower than in wild-type mice. After induction, the HOMA-β significantly decreases in both wild-type and uPA-/- mice. ** *p* < 0.01 (Kruskal–Wallis H test).

Twenty-two wild-type and 12 uPA-/- BALB/c mice received induction of T2DM. While half of the wild-type mice developed T2DM within one week, at a success rate of 50%, all the uPA-/- mice developed T2DM (success rate: 100%). The uPA-/- mice developed significantly higher hyperglycemia after induction, and the level difference persisted for one month (Figure 1B). The uPA-/- mice non-significantly lost a little weight in the development of hyperglycemia, which may have been due to hyperglycemia-related polyuria and mild dehydration (Figure 1C). These above results suggest that uPA-/- mice are prone to T2DM.

Interestingly, the Immunohistochemical (IHC) staining of uPA on the islet of wild type showed that the uPA expression on islet declined remarkably on D3 (DX means X days after successful development of T2DM) after induction (Figure 1D). The data indicate that hyperglycemia was associated with the suppression of uPA expression on islet.

### 2.2. uPA-/- Mice Failed to Regain Normal Insulin Secretion and Regeneration

**Figure 2:**
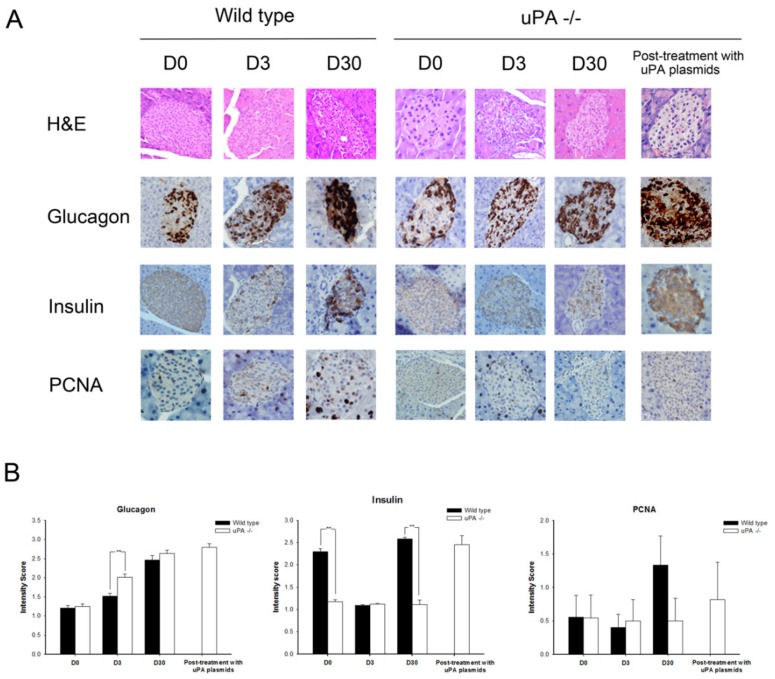
Hematoxylin and eosin (H&E), and immunohistochemical (IHC) stain of glucagon, insulin, and proliferating cell nuclear antigen (PCNA) on islet before and after induction of type 2 diabetes mellitus in wild-type and uPA-/- mice and after treatment with uPA plasmids in uPA-/- mice. (**A**) The H&E stain of islet showed no pathologic structural change in either wild-type or uPA-/- mice. The expression of glucagon on islet increased after induction both in wild-type and uPA-/- mice. Obviously, insulin particles of islet 30 days after induction (D30) increased in wild-type mice as compared with uPA-/- mice. Meanwhile, the PCNA expression of islet increased on D30 in wild-type mice. After treatment with uPA plasmids in uPA-/- mice, the glucagon, insulin, and PCNA expressions are similar to those on D30 in wild-type mice. (**B**) The quantitative intensity score of expression of glucagon, insulin, and PCNA on islets on induction day (D0), D3, and D30, after induction was assessed and scored by pathologists. The intensity scores of glucagon in uPA-/- mice were significantly higher than those in wild-type mice on D3. The intensity scores of insulin in wild-type mice were significantly higher than those in uPA-/- mice on D3 and D30. The intensity score of PCNA in wild-type mice on D30 was higher, but did not reach statistical significance. ** *p* < 0.01 (Kruskal–Wallis H test).

Figure 1E–H shows the changes in insulin, glucagon, insulin resistance, and insulin secretion during the course of T2DM development. It was predictable that insulin resistance in the mice increased with the feeding of a high-fat diet. The insulin levels and Homeostasis model assessment-insulin resistance (HOMA-IR) increased progressively in the wild-type mice, whereas glucagon levels, the reciprocal hormone of insulin, declined gradually with elevation of insulin. While Homeostasis model assessment-β (HOMA-β) was significantly lower on D3 in wild-type mice, the HOMA-β, however, increased mildly on D30, although it did not reach a significant difference. We hypothesized that the insulin secretion in wild-type mice might be revived partly after streptozotocin (STZ) destroyed β cells. On the other hand, the increments of insulin levels and HOMA-IR in uPA-/- mice were lower than in wild-type mice, and the glucagon levels in uPA-/- mice were higher than in wild-type mice. However, it was noteworthy that the same induction protocol on uPA-/- mice caused the HOMA-β to decline abruptly on D3 and remain low until D30, where the D30 value was even lower than that of D3. The data suggest that the β cells of uPA-/- mice failed to regenerate after being destroyed by STZ. In the pathologic morphology (Figure 2A), the IHC stain of insulin in the wild-type mice showed the insulin particles increased obviously on D30. A similar finding was not noted in uPA-/- mice, indicating less β cell mass as compared with the wild-type mice. After quantitative analysis (Figure 2B), the intensity scores of insulin particles in islet of wild-type mice were significantly higher than those in uPA-/- mice on D0 and D30. The proliferating cell nuclear antigen (PCNA) of islet in wild-type mice on D30 increased mildly, although it did not reach statistical significance. This demonstrates that the regeneration of β cells after injury is poor in uPA-/- mice. Furthermore, this accords with lower levels of insulin secretion in uPA-/- mice on D30 (Figure 1H).

### 2.3. After Treatment with uPA Plasmid, uPA-/- Mice Were Less Likely to Develop T2DM

**Figure 3:**
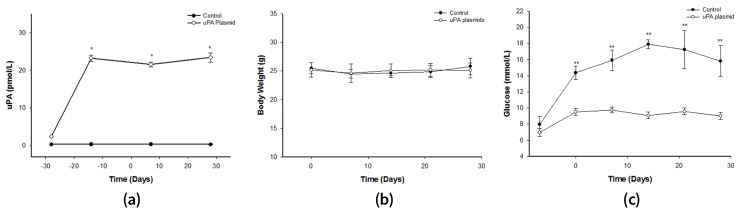
Changes in uPA, body weight, and blood glucose in uPA-/- mice treated with uPA plasmids. The uPA-/- mice were treated with intramuscular injection of uPA plasmids (blank circle) or control plasmid (black circle) weekly. The uPA, body weight, and blood glucose were measured (n = 6). (**A**) The uPA levels significantly increased after treatment with uPA plasmid in uPA-/- mice. (* *p* < 0.05) (**B**) Body weight during the whole experiment showed no significant change in the two groups. (**C**) Blood sugar did not elevate after induction, but mice injected with control plasmid developed hyperglycemia. In other words, no uPA-/- mouse developed T2DM after treatment with uPA plasmids (Mann–Whitney U test).

After the injection of uPA plasmids in uPA-/- mice, the uPA level was significantly higher than in those injected with control plasmid, and remained steady until the end of the study (Figure 3A). The most interesting observation was that not one mouse developed T2DM after STZ/Nicotinamide (NTM) induction (Figure 3C). Conversely, all uPA-/- mice injected with control plasmids developed T2DM. Accordingly, the expressions of insulin and PCNA on islet in plasmid-treated uPA-/- mice were found to have increased as in the wild-type mice (Figure 2A). Based on these findings, we presumed that the ability to regenerate β cells in uPA-/- mice seemed to have recovered after supplementation of uPA plasmid.

### 2.4. The uPA Secretion Rate Decelerated and uPA Expression on β Cells Declined in High-Glucose Condition

**Figure 4:**
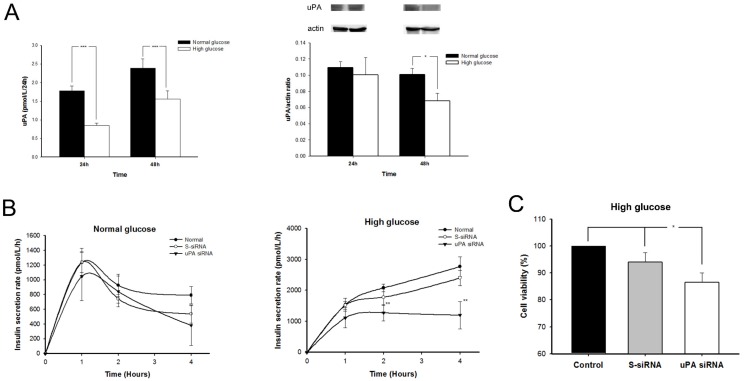
The (**A**) uPA secretion rate and expression on β cells; (**B**) insulin secretion rate, in normal glucose or high glucose; and (**C**) β cell proliferation in high glucose (n = 6). (A) The uPA secretion rate of β cells in normal glucose (black bar) was significantly faster than those in high glucose (white bar) treated for both 24 and 48 h. *** *p* < 0.001. The uPA expression on β cells significantly declined after treatment with high glucose for 48 h. * *p* < 0.05 (Mann–Whitney U test) (B) The insulin secretion rate in normal glucose showed no significant difference among control (black circle), S-siRNA (blank circle) or uPA siRNA (inversed triangle) groups. After silencing uPA by siRNA, the insulin secretion rate of β cells decelerated significantly in high glucose. ** *p* < 0.01. (Kruskal–Wallis H test) (C) β cell proliferation is evaluated by MTT assay while incubated in high glucose for 48 h. After silencing uPA expression by siRNA (white bar), the β cell proliferation significantly decreased in high glucose as compared with control (black bar) and S-siRNA groups (gray bar). * *p* < 0.05 (Kruskal–Wallis H test).

The uPA secretion rate and uPA intra-cellular expression of β cells after treatment with normal or high glucose are shown on Figure 4A. Comparing with normal glucose, uPA secretion rate of β cells, adjusting for cellular proliferation (3-(4,5-cimethylthiazol-2-yl)-2,5-diphenyl tetrazolium bromide (MTT)), significantly decelerated in high glucose for both 24 and 48 hour (h). In addition, the uPA intra-cellular expression in β cells also significantly decreased in high glucose for 48 h. These findings accorded with the result in the in vivo mice model (Figure 1D).

### 2.5. Insulin Secretion Rate Decelerated and Replication of β Cell Declined in High Glucose after Silencing uPA

The role of uPA in insulin secretion was examined in a uPA-silenced β cell model (Figure 4B). In normal glucose condition, the rate of insulin secretion in mouse β cells peaks in the first hour and then falls gradually, with no significant difference among normal, scramble siRNA (S-siRNA), and uPA siRNA groups. In high-glucose condition, the insulin secretion rate rapidly rises in the first hour, and then progressively accelerates with time in normal and S-siRNA groups. However, in the uPA siRNA group, the rate of insulin secretion in the first hour was lower than in normal and control groups. The insulin secretion rate in uPA siRNA group did not accelerate and was significantly lower than in the normal and S-siRNA groups. The results indicate that a deficiency of uPA in β cells impaired the insulin secretion in high-glucose condition, suggesting that uPA may contribute to regulating insulin secretion of β cells after high-glucose stimulation.

To assess the role of uPA in β cell regeneration, the comparison of β cell proliferation in normal, S-siRNA, and uPA siRNA groups was studied by MTT assay (Figure 4C). The β cell proliferation was significantly inhibited after uPA silencing by siRNA, while cell proliferation was inhibited mildly in the S-siRNA group. In eliminating the chemical effect caused by the transfection reagent, our result clearly indicated that a deficiency of uPA could lead to the impairment of β cell regeneration.

### 2.6. Treatment with uPA Enhanced β Cell Proliferation in Normal Glucose, but Insulin Secretion Rate Decelerated in High Glucose after Treatment with Anti-uPA Antibody

**Figure 5:**
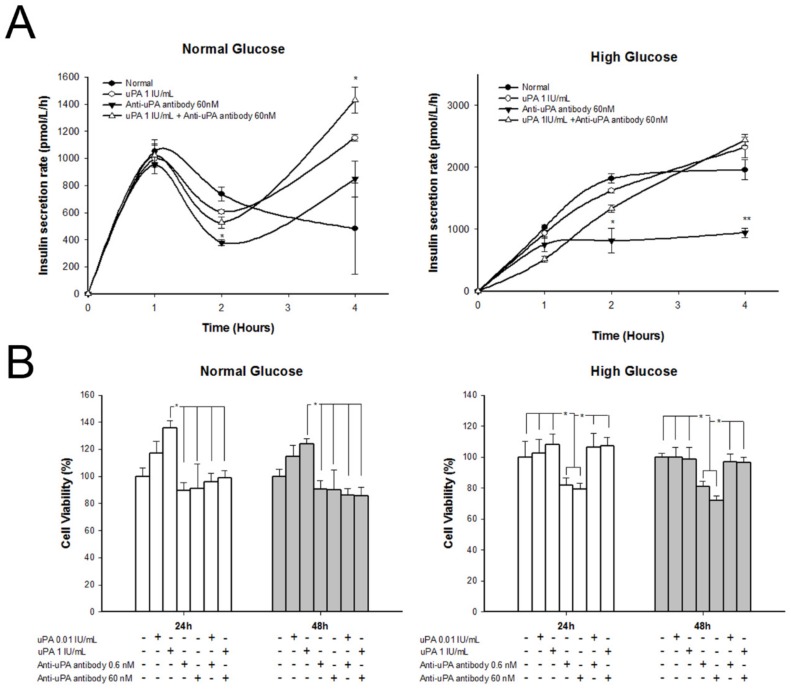
Insulin secretion rate and β cell proliferation in normal glucose or high glucose after treating with uPA or anti-uPA antibody. (**A**) Insulin secretion rate of β cells treated with control (black circle), uPA (blank circle), anti-uPA antibody (inverted black triangle), or uPA + anti-uPA antibody (blank triangle). In normal glucose, the insulin secretion rate of β cells with treating uPA antibody significantly decreased at 2 h. However, the insulin recreation rate at 4 h significantly increased after treating uPA and co-treating uPA and uPA antibody. The insulin secretion rate of β cells significantly decelerated in high glucose, after being treated with uPA antibody. * *p* < 0.05, ** *p* < 0.01 as compared with control group (Kruskal–Wallis H test). (**B**) Proliferation of β cells treated with uPA, anti-uPA antibody, or uPA + anti-uPA antibody for 24 or 48 h. The β cell proliferation increased after being treated with uPA in normal glucose. In high glucose, β cell proliferation decreased after being treated with anti-uPA antibody. * *p* < 0.05 (Kruskal–Wallis H test).

The insulin secretion rate and cell proliferation of β cells after treatment with different concentrations of uPA and anti-uPA antibody is shown in Figure 5. The insulin secretion rate of β cells significantly decelerated after treatment with uPA antibody in high glucose, at 2 h, and persisted for 4 h. In normal glucose, the insulin secretion rate of β cells treated with anti-uPA antibody declined in the second hour, but was restored thereafter. In addition, the insulin secretion rate at 4 h significantly increased after treating uPA and co-treating uPA and uPA antibody. The finding was similar to the results for β cells with silenced uPA. However, β cells treated with high levels of uPA did not show an accelerated insulin secretion rate in either normal glucose or high glucose. In addition, cell proliferation of β cells significantly increased after being treated with uPA in normal glucose. In addition, the effect of treatment was dose-dependent. In high glucose, β cells treated with uPA did not significantly increase proliferation. However, β cells treated with anti-uPA antibody showed inhibited cell proliferation in high glucose.

### 2.7. Better Insulin Secretion Capability after Oral Glucose Challenge in Patients with T2DM and Higher uPA Levels

**Figure 6:**
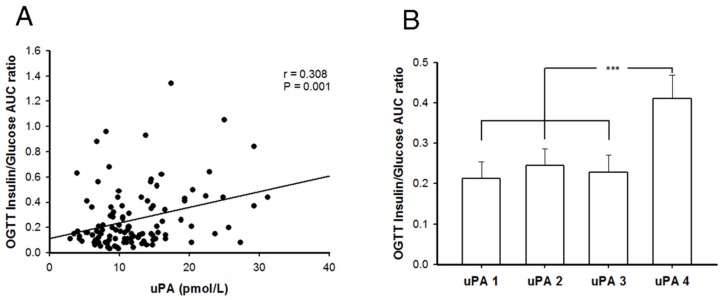
The correlation between uPA and insulin secretion in patients with T2DM. (**A**) The uPA levels showed a weak and significant linear relationship with insulin secretion in patients with T2DM. (r = 0.308, *p* = 0.001; Pearson’s correlation) (**B**) Patients with T2DM were divided into quartiles according to uPA level. The patients with the highest quartile of uPA (uPA4) showed better ability of insulin secretion than those in the other three groups (uPA1-3). However, there was no significant difference among the other three groups. *** *p* < 0.001 (One-way ANOVA with Bonferroni post hoc test).

There were 112 subjects with T2DM enrolled in our study, and they were divided into four quartile groups based on their plasma uPA levels, as shown in Table 1. Their general characteristics among each uPA quartile group are shown in Table 1. The uPA levels between different groups showed a significant difference statistically. While the HgbA1c of groups uPA4 and uPA2 were significantly lower than those of uPA1 and uPA3, the fasting plasma glucose of group uPA4 was significantly lower than that of uPA3, and LDL of group uPA4 was significantly higher than that of uPA1. C-peptide, adiponectin and free fatty acid showed no significant difference between quartiles of uPA groups. However, C-peptide in uPA4 groups was non-significantly higher than in the other three groups. The correlation between the uPA and insulin-to-glucose area under curve (AUC) ratio of all subjects is demonstrated in Figure 6A, which shows a significant linear relationship (r = 0.308). After adjusting for age, sex, and BMI, uPA is also independently and significantly associated with insulin-to-glucose AUC ratio (β = 0.289, 95% Confidence Interval: 0.128–0.604, *p* = 0.003). This result indicates a weak relationship between uPA levels and insulin secretion ability for patients with T2DM. After dividing the subjects into four quartiles groups according to their uPA levels, it was found statistically significant that subjects with the highest level of uPA had better insulin secretion ability than the three lower uPA groups (Figure 6B). Based on the clinical data, we again ascertained that uPA level is associated with insulin secretion in T2DM, especially for men with a higher uPA level.

### 2.8. Discussion

According to the existing literature and our data, BALB/c mice are refractory to induction of T2DM, using a high-fat diet and STZ/NTM. In our study, the uPA-/- BALB/c mice were sensitive to induction of T2DM. However, after treatment with intra-muscular uPA plasmids, uPA-/- BALB/c mice were again resistant to induction of T2DM. In the IHC stain of insulin and PCNA, the islet of uPA-/- BALB/c mice after induction of T2DM showed poor regeneration. In our in vitro study, we found a deceleration of the insulin secretion rate and decline in replication of the β cell line in high-glucose condition after silencing uPA by siRNA. After treatment with uPA, β cell proliferation increased in normal glucose. After blocking circulating uPA by anti-uPA antibody, the insulin secretion rate decelerated in high glucose, and β cell proliferation declined in high glucose. At the same time, hyperglycemia also suppressed uPA expression in β cells in BALB/c mice and in the in vitro cell model. Clinical subjects with higher levels of uPA had a better ability to secrete insulin. Our results indicated that uPA is an important factor in insulin secretion and β cell regeneration, and deficiency of uPA may contribute to development of T2DM.

As compared with C56BL/6 mice, Hayashi K et al. found BALB/c mice needed a larger dose of STZ for induction of diabetes [15]. Similarly, BALB/c mice had a lower fasting-blood glucose level and increment of blood glucose after being fed a high-fat diet [16]. According to the results, BLAB/c mice with increased insulin resistance after being fed a high-fat diet had better compensative ability to secrete more insulin for maintaining glycemic homeostasis. These findings could explain the lower induction rate in our BALB/c mice. It is most interesting that the induction rate of T2DM in BALB/c mice abruptly elevated after their uPA gene was knocked out. This suggests that uPA is an important factor in the development of T2DM, which motivated us to undertake this study for underlying causes.

The results of our in vivo study showed uPA-/- mice had lower HOMA-β and HOMA-IR—which oppose each other in the development of hyperglycemia—than did the wild-type mice before and after induction of T2DM. However, the uPA-/- mice after induction had higher hyperglycemia. It is reasonable to suggest that a deficiency of uPA has a more prominent effect on insulin secretion than on insulin resistance, resulting in more hyperglycemia in uPA-/- mice. The impact of uPA deficiency on insulin secretion was also demonstrated in our in vitro cell model. The rate of insulin secretion in the uPA silenced β cell line decelerated in high-glucose condition, which was consistent with the findings in our in vivo study. The explanation may be that insulin particles increase in islet, but HOMA-β did not increase on D30 in wild-type mice because HOMA-β was not evaluated after the glucose challenge. Similar results were seen in cellular model. We found that the insulin secretion rate in normal glucose decreased at 2 h in uPA antibody group, and then mildly increased at 4 h. In high glucose, the insulin secretion rate significantly decreased at both 2 and 4 h. In human investigation, uPA is positively related to the ability for of insulin secretion during oral glucose tolerance test (OGTT). These results may verify our presumption that the uPA contributes to insulin secretion in high glucose. However, the exact mechanism by which uPA affects insulin secretion in high glucose is still unclear. Christow et al. reported that uPA stimulates intracellular calcium release by activating inositol 1,4,5-trisphosphate, a crucial signal in insulin secretion of β cells after glucose stimulation, in human promyelocytic cells [17]. However, there is no study that has shown a similar mechanism in β cells.

The apoptosis of β cells over regeneration may be the rationale for impaired insulin secretion in development of T2DM. Our in vivo and in vitro studies indicated that deficiency of uPA is associated with decreased regeneration of β cells. The most potent evidence is that insulin particles of the islet increased in wild-type mice one month after injury of insulin-producing β cells, by injection of STZ. Similarly, Bonner-Weir et al. found euglycemia had developed, and observed partial β cell regeneration, 10 days after they STZ-induced diabetic mice [18]. Thereafter, several studies explored the mechanisms of β cell regeneration for potential therapy in T2DM [19,20]. However, a similar result was not found in uPA-/- mice. The proliferating biomarker PCNA in islet of wild-type mice on D30 showed a nonsignificant increase, which may be due to wide variation by individual subjective judgement. However, we found a decrease of cellular proliferation was noted in the β cell line after silencing uPA by siRNA and treatment with anti-uPA antibody in high glucose, which supports our findings in the mouse model. In addition, treatment with uPA in the in vitro study increased β cell proliferation in normal glucose, but not in high glucose. Generally, persistent hyperglycemia would induce glucotoxicity, which inhibits β cell replication. However, we found that treatment with uPA neutralizes β cells proliferation in our in vitro study. Meanwhile, the effect of glucotoxicity inhibited β cell proliferation amplified by treatment of anti-uPA antibody in high glucose. These concordant results in in vivo and in vitro studies emphasize the important role of uPA in β cell proliferation and the close relationship between lack of uPA and development of T2DM. The pathophysiology of uPA in β cell regeneration is still unclear. Some studies showed uPA contributed to regeneration in some kinds of cells. Shimizu et al. found that regeneration of hepatocyte was impaired in plasminogen activator-deficient mice, which is associated with activation of the hepatocyte growth factor [21]. Moreover, uPA also contributes to the regeneration of skeletal muscle cells by increasing the hepatocyte growth factor [22,23]. However, our study is the first paper identifying the role of uPA in β cell regeneration. Teramura et al. found uPA modified the surface of the transplanted islet and increased β cell survival, and suggested that the fibrolytic effect of uPA improved blood clot-related inflammation [14]. It is possible that a uPA-modified surface on β cells may contribute to cellular regeneration.

Also noteworthy is that uPA-/- mice were treated with uPA plasmid, and their uPA levels elevated subsequently. No uPA-/- mice receiving uPA plasmids developed hyperglycemia. On the other hand, all uPA-/- mice receiving control plasmid developed hyperglycemia after induction. The β cell regeneration of the islet in treated mice resembled that of wild-type mice. In addition, IHC stains of insulin and glucagon on islet in uPA-/- mice treated with uPA plasmid showed a simultaneous increase, which may imply that increasing apoptosis and regeneration of β cells on effects of uPA-/- and uPA plasmids supplementation at the same time led to euglycemic homeostasis in mice. This interesting finding suggests that failure of β cell regeneration might be reversible after supplementation of uPA. Although the treatment of uPA has not yet been applied in clinical studies, our findings may provide a new direction in the use of uPA for prevention of T2DM in the future.

**Figure 7:**
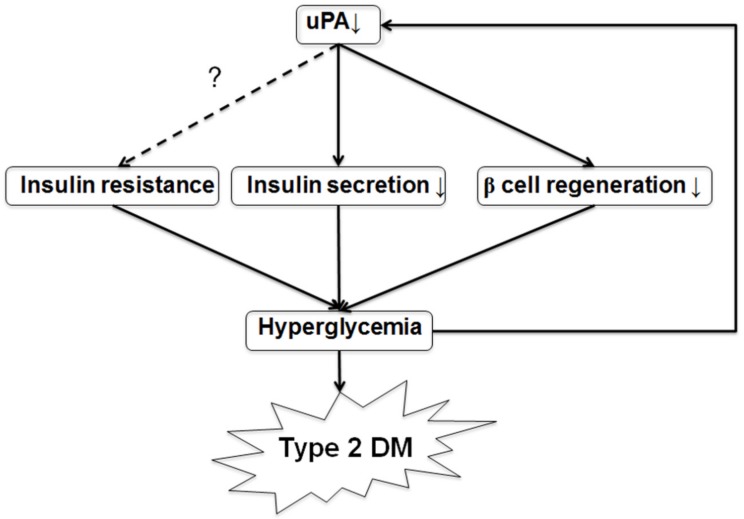
A model of uPA deficiency leading to development of T2DM. According to our results, we propose a possible model of development of T2DM following uPA deficiency. Numerous genetic and environmental factors contribute to uPA deficiency in some people. The uPA deficiency, in turn, contributes to both insulin secretion impairment and poor β cell regeneration. These two factors, plus insulin resistance, lead to hyperglycemia. Hyperglycemia suppresses uPA expression in islet. The vicious cycle of β cell failure is established on uPA deficiency. Finally, T2DM develops, and patients will need insulin supplementation eventually. Thus, our model might point the way for early detection and treatment of T2DM.

We also found uPA expression on the islet was reduced in wild-type mice after development of T2DM. However, there has been no study exploring the uPA expression in β cells in T2DM. Fisher EJ et al. found that uPA levels and activity declined in mesangial cells in high-glucose condition [11]. According to our results, we presumed that hyperglycemia after induction may suppress uPA expression on islet. Using the above findings, we delineated the possible model of β cell failure and development of T2DM (Figure 7). Uncertain genetic or environmental factors may contribute to uPA deficiency. The deficiency of uPA impairs insulin secretion and β cell regeneration, and it promotes hyperglycemia. With the superimposition of insulin resistance, hyperglycemia becomes more severe. Then, hyperglycemia suppresses uPA expression in β cells. Consequently, a vicious cycle of T2DM results from the initial uPA deficiency. Furthermore, the vicious cycle leads to progressive β cell mass loss, so that patients with T2DM eventually need insulin supplement for glycemic control.

In our study, the relationship between uPA and insulin secretion in mice and cell models was identified by clinical exploration. In clinical investigation of patients with T2DM, we found that uPA levels were mildly positively related to insulin secretion after the oral glucose challenge. From further analysis, the patients in the highest quartile of uPA had significantly better insulin secretion than did the three lower quartile groups. The relationship suggested a threshold of uPA level for better insulin secretion. If the uPA level is above the threshold, the patient’s insulin secretion would be optimal. Nevertheless, optimal insulin secretion needs to be defined. Our clinical results suggest a novel direction for administration of uPA for treatment of patients with T2DM or prevention of β cell failure. However, several studies also showed uPA is linked to cancer invasion and metastasis [24,25]. Supplement of overdose of uPA may increase the risk of potential cancer spreading. Thus, the adequate balance of uPA supplementation needs further study.

The receptor of uPA, urokinase plasminogen activator receptor (uPAR), is composed of three extracellular domains (D1, 2, and 3) and may be shed from the cell surface by several protease as a soluble bioactive peptide in blood and body fluid (soluble uPAR) that has multiple biological properties. We had analyzed the soluble uPAR in health subjects and patients with T2DM in a previous study. [26] However, the levels of soluble uPAR between healthy subjects and T2DM patients without nephropathy showed no significant difference. However, a large bundle of studies showed soluble uPAR is associated with various complications of T2DM, such as nephropathy, retinopathy, peripheral artery disease, and cardiovascular events [26,27,28,29]. Nonetheless, whether deficiency of uPA and high levels of uPAR synergistically contribute to insulin secretion impairment and complications of T2DM needs further explorations in the future.

There were some limitations in our study. First, we did not have the pathology of human islet for testing our theory in clinical subjects. The relationship between uPA and β cell regeneration in human subjects is still unknown. However, the oral glucose test for evaluation of insulin secretion is commonly used in clinical research worldwide. Second, the mechanisms of uPA in insulin secretion and β cell regeneration are still unknown. One possible explanation may be associated with activation of the hepatocyte growth factor [21,22,23] by uPA, but it is still not a reasonable explanation for the real mechanism of β cell regeneration. In this study, we explored the influence of uPA on development of T2DM. Detailed cellular pathophysiology of uPA in β cells requires further study. Moreover, we used MTT assay, which evaluates mitochondrial dehydrogenase activity, for assessing cellular proliferation and apoptosis. However, the result was in line with our in vivo study. In addition, some studies also used MTT assay for assessing cellular proliferation or apoptosis [30,31]. Thus, it did not reduce our valuable contribution. Finally, we enrolled subjects with T2DM for clinical investigation. There are many factors affecting the glycemic and lipid control, such as diet control, exercise, and drug compliance. Thus, there were some variations between groups of uPA quartile. The relationship between uPA and insulin secretion in healthy subjects needs to be analyzed in the future.

## 3. Materials and Methods

### 3.1. In Vivo Mice Study

#### 3.1.1. Developmental of uPA-/- BALB/c Mice

##### Backcross Breeding

In this study, the B6.129S2-Plautm1Mlg/J mouse strain (Jackson Laboratory; GA, USA) was backcrossed onto a pure BALB/cByJNarl (the National Laboratory Animal Breeding and Research Center; Taipei, Taiwan). The first cross was between the female BALB/cByJNarl (recipient inbred partner) and male B6.129S2-Plautm1Mlg/J that carried the donor allele. Then, the procedure was repeated for at least five to ten generations backcross cycles before the experiment, since a strain was considered a congenic incipient after five to nine backcross cycles (N5-N9). The mice used in the present study were at least N5 generation or greater. The identification of genotype was confirmed by polymerase chain reaction.

##### DNA from Tail Biopsy and Genotyping Identification

A piece of murine tail (~0.5mm) was cut and placed in a polypropylene microfuge tube when the mouse was 3–4 weeks old. After incubating with DNA digestion buffer and proteinase K at 55 °C overnight, the supernatant was collected by centrifugation at 13,500 rpm for 15 min, followed by precipitating and washing the murine DNA by absolute and 75% ethanol at 4 °C, respectively. The obtained DNA was dried at room temperature, and then dissolved in sterilized water prior to analysis. The genotyping of uPA-/- was identified by polymerase chain reaction and genetic sequence identification, respectively. The primer sequences (5′ to 3′) were CCGGTTCTTTTTGTCAAGACCG, CGGCAGGAGCAAGGTGAGAT, TCTGGAGGACCGCTTATCTG, and CTCTTCTCCAATGTGGGATTG.

#### 3.1.2. Induction of T2DM Mouse Model

The male wild-type and uPA-/- BLAB/c mice were fed a high-fat diet (40% fat, Research Diets Inc., NJ, USA) from the age of 5 weeks. NTM (200 mg/Kg) was injected intraperitoneally 15 min before the first dose (75 mg/Kg) of intravenous STZ. Five days later, the mice received the second dose of NTM and STZ [32,33]. Murine blood sugar and glucosuria were measured daily. The successful induction of T2DM was defined as blood sugar more than 11.1 mmol/L (200 mg/dL) and urine-strip color presenting four positives simultaneously. Groups of wild-type and uPA-/- BALB/c mice were euthanized before induction at D0, D3, and D30 after occurrence of T2DM. Blood samples were collected before euthanasia and the pancreatic tissues were harvested after euthanization. The experimental animal protocol was approved by the Institutional Animal Care and Use Committee at the National Defense Medical Center, Taipei, Taiwan (IACUC12-058), and performed in accordance with the relevant guidelines and regulations.

#### 3.1.3. uPA-/- Mice Treated with uPA Plasmid

The uPA-/- BALB/c mice received weekly intramuscular injections of purified uPA plasmids (100 mg) or control plasmids from three weeks before induction of T2DM. The levels of uPA were measured weekly. Mice having successful uPA boost and T2DM induction were subjected to all the foregoing tests, including the induction rate.

#### 3.1.4. Measurement of Murine uPA, Insulin, Glucagon, Insulin Resistance, and Secretion

The plasma and serum were separated from blood by centrifugation within one hour of being drawn and were stored at −80 °C, prior to analysis. The uPA was measured, using the Mouse uPA Total Antigen Assay enzyme-linked immunosorbent assay (ELISA) kit (Molecular Innovations, Novi, MI, USA). The serum insulin was measured, using the Mouse Insulin ELISA Kit (Mercodia AB, Uppsala, Sweden) with the intra- and inter-assay coefficients of variation at 3.4% and 3.6%, respectively. The serum glucagon was measured, using Human/Mouse/Rat Glucagon ELISA Kit (RayBiotech, Norcross, GA, USA), which has the intra- and inter-assay coefficients of variation of <10% and <15%, respectively. HOMA-IR and -β were measured to assess insulin resistance and insulin secretion ability, respectively [34].

#### 3.1.5. Hematoxylin and Eosin (H&E) and IHC Stains of Islet for Insulin, Glucagon, uPA, and PCNA

The pancreatic tissue was fixed in 10% formaldehyde fixative solution and embedded in paraffin. Sections of formalin-fixed pancreatic tissue were immersed in xylene for 5 min, three times, to remove paraffin, followed by rehydration in graded ethanol and stained with H&E.

For IHC staining of insulin in islet, the slices were incubated with 1:500 dilution of the primary antibody, rabbit anti-insulin antibody (Cell Signaling Technology, Danvers, MA, USA), at room temperature, for 1 h, after removing paraffin and rehydrating. Then, the slices were incubated with 1:200 dilution of secondary antibody, goat anti-rabbit IgG-HRP (Invitrogen, Life Technologies, Grand Island, NJ, USA), for 1 h before wash. The stain was visualized by use of BAD chromogen.

For IHC staining of glucagon in islet, the procedure was the same as above, except that anti-mouse glucagon antibody (1:5000; Abcam, Cambridge, MA, USA) was used as the primary antibody and goat anti-mouse IgG-HRP (1:200; Santa Cruz Biotechnology, Santa Cruz, CA, USA) was the secondary antibody. For IHC staining of PCNA in islet, the rabbit anti-PCNA (1:200; Santa Cruz Biotechnology) was the primary antibody and the goat anti-rabbit IgG-HRP (1:200; Invitrogen, Life Technologies) was the secondary antibody. For IHC staining of uPA in islet, the anti-mouse uPA antibody (1:200; Abcam) was the primary antibody, and the goat anti-mouse IgG-HRP (1:200; Santa Cruz Biotechnology) was the secondary antibody. Slides were examined for development of fluorescence, using an optical photomicroscope (Olympus Corporation, Tokyo, Japan), and for quantitative analysis. At least 5 islets per slide were examined and scored by at least two well-trained pathologists for positively stained areas. For every islet examined, a score of 0 to 4 (0, no positive staining; 1, minor staining; 2, moderate staining; 3, prominent staining with expanded positively stained areas; 4; prominent staining of large areas) was given.

### 3.2. In Vitro Cell Study

#### 3.2.1. Assessing uPA Expression and Secretion of Murine β Cells in Normal or High Glucose Medium

Murine pancreatic β cells, NIT-1 cells (American Type Cell Culture, Manassas, VA), were seeded in culture platelet and treated with normal (7 mM) or high (25 mM) glucose culture medium for 24 or 48 h, respectively. Culture media from each well were collected, and uPA levels were measured by Mouse uPA Total Antigen Assay ELISA kit (Molecular Innovations, Novi, MI). Thereafter, equal amounts of protein (30 μg) from each well, after harvesting cells, were separated by gel. The gel was electroblotted onto a nitrocellulose membrane, and then incubated with 1:1000 dilutions of anti-uPA antibody (Abcam, Cambridge, MA), at 4 °C overnight. After incubation in horseradish peroxidase-conjugated anti-rabbit antibody, the membrane-bound antibody detected was incubated with Western blot detection system and captured on X-ray film.

#### 3.2.2. Measurement of Insulin Secretion of β Cells with and without uPA Silencing

NIT-1 cells were prepared and mixed with 0.4 μL of TurboFect transfection reagent (Thermo Fisher Scientific Inc., Waltham, MA, USA) containing 5 nM of siRNA (Santa Cruz biotechnology), and then they were seeded in platelet for 8 h. For the control purpose, equal amounts of S-siRNA were used.

NIT-1 cells with and without uPA silencing were first incubated with 10% fetal bovine serum and 25 mM of glucose, for 40 h, to exhaust intracellular insulin. Then, the NIT-1 cells were stabilized with normal glucose (7 mM) culture medium for 1 h. Subsequently, the NIT-1 cells were treated with culture medium containing 25 mM or 7 mM of glucose, respectively. The insulin levels in culture media were measured at 1, 2, and 4 h.

#### 3.2.3. Measurement of β Cell Proliferation with and without uPA Silencing in High Glucose

NIT-1 cells with and without uPA silencing were incubated in a high-glucose (25 mM) cell medium for 40 h. Then, 10 mL of 5 mg/mL MTT solution (Invitrogen, Life Technologies) was added to each well for labeling live cells. After incubation, the spectrometric absorbance at 570 nm was used for reading cell viability.

#### 3.2.4. Assessment of Insulin Secretion Rate and β Cell Proliferation after Treating with uPA

NIT-1 cells were incubated with normal (7 mM) or high glucose (25 mM), respectively. The first group of NIT-1 cells was treated with 0.01 IU/mL or 1 IU/mL of uPA (Yao Chih Hsiang Inc., Taiwan) in culture medium. The second group of NIT-1 cells was treated with 0.6 nM or 60 nM of anti-uPA antibody (Abcam, Cambridge, MA, USA) in culture medium. The third group of NIT-1 cells was treated with uPA and uPA antibody at the same time. Insulin secretion rate and cell proliferation of β cells were measured (method as described above).

### 3.3. Clinical Investigation

#### 3.3.1. Subjects

Subjects aged 20–70 years who had been diagnosed with T2DM within 5 years, from a local teaching hospital in Taiwan, were enrolled in the study. Subjects who had a history of major medical diseases, including coronary heart disease, myocardial infarction, stroke, renal failure, or type 1 diabetes were excluded. Three days prior to the study, subjects were asked to maintain a stable diet. On the day of the study, subjects visited the clinic at 8 a.m. after a ten-hour fast. A complete physical examination was done, and body mass index was calculated as weight/height^2^ (kg/m^2^). Blood pressure was measured by nursing staff, using standard mercury sphygmomanometers on the right arm of seated participants. The study protocol was approved by the institutional review board and ethics committee, Cardinal Tien Hospital, Xindian, New Taipei City, Taiwan (IRB Approval Number: CTH-97-2-4-035), and performed in accordance with the relevant guidelines and regulations. Written informed consent was obtained from all study subjects.

#### 3.3.2. OGTT

On the study day, an intravenous catheter was placed in the antecubital vein. Fasting-blood samples were drawn for biochemistry analysis. Subjects orally consumed a standard 75 g dose of glucose. Plasma glucose and insulin concentrations were measured before and 5, 10, 20, 30, 45, 60, 80, 100, 120, and 180 min after the oral glucose challenge.

#### 3.3.3. Laboratory Measurement

Human plasma and serum were separated from blood within 1 h of blood collection and were frozen at –80 °C, until analysis.

Insulin was measured by the Coat-A-Count solid-phase radioimmunoassay kit (Diagnostic Products, Los Angeles, CA). Intra- and inter-assay coefficients of variance for insulin were 3.3% and 2.5%, respectively. Plasma glucose was measured by using a YSI 203 glucose oxidase analyzer (Yellow Spring Instrument, Yellow Spring, OH, USA). Serum triglyceride was measured based on a dry multilayer slide method by the Fuji Dri-Chem 3000 analyzer (Fuji Photo Film, Tokyo, Japan). Serum low-density lipoprotein-cholesterol and high-density lipoprotein-cholesterol levels were determined by an enzymatic cholesterol assay after dextran sulphate precipitation. The levels of glycated hemoglobin A1c were evaluated using ion-exchange high-pressure liquid chromatography (Bio-Rad Variant II, Hercules, CA, USA). The C-peptide was measured by C-peptide ELISA kit (10-1141-01, Marcodia). Intra- and inter-assay coefficients of variance for insulin were 4.3% and 6.2%, respectively. Adiponectin was measured by Adiponectin Dutset (DY1065, R&D systems, Minneapolis, MA, USA). Intra- and inter-assay coefficients of variance for insulin were 4.3% and 6.2%, respectively. Free fatty acid was measured by free fatty acid ELISA kit (KA1667, Abnova). Inter-assay coefficients of variance for insulin was 2.23%. The human uPA concentrations were measured by the Human uPA ELISA kit (R&D systems, Minneapolis, MA, USA) in duplicate.

### 3.4. Statistical Analysis

SPSS version 13.0 statistical package for Windows (SPSS, Chicago, IL, USA) was used for data analysis. The continuous variables were expressed as mean ± SE. In animal and cell models, nonparametric statistics with Mann–Whitney U test were used for comparison of two groups. For comparison of more than two groups in animal or cell studies, nonparametric statistics with Kruskal–Wallis H test were applied. The ratio of AUC in insulin and glucose during OGTT was calculated and represented as ability of insulin secretion after glucose challenge. For data analysis, all subjects with T2DM were divided into four quartiles, uPA1–uPA4, from the lowest to highest, based on plasma uPA levels. The correlation between the uPA and insulin/glucose AUC ratio was evaluated with Pearson’s correlation. The independent factor of uPA on insulin/glucose AUC ratio was assessed by multivariate linear regression, after adjusting for age, sex, and BMI. One-way ANOVA with Bonferroni post hoc test was applied, in order to compare the differences among quartiles. All statistical data were expressed as two-sided, and *p*-values less than 0.05 were considered to be statistically significant.

## 4. Conclusions

In conclusion, a lack of uPA impairs insulin secretion and regeneration of β cells in mice and cell models and induces hyperglycemia. Hyperglycemia reduces the uPA expression on the islet, resulting in a vicious cycle that promotes the development of T2DM. Patients with lower uPA levels have a reduced ability of insulin secretion after the oral glucose challenge. A link between the deficiency of uPA and the developmental of T2DM is suggested, offering a new direction for the study and treatment of T2DM. The role of uPA is an important factor in the development of T2DM, and further study of these mechanisms is needed.

## Figures and Tables

**Table 1:** The general characteristics of subjects with type 2 diabetes mellitus in different groups.

	uPA1	uPA2	uPA3	uPA4	All
n	28	28	28	28	112
uPA (pmol/L)	6.1 ± 1.4^2,3,4^	9.5 ± 0.8^1,3,4^	12.9 ± 1.3^1,2,4^	20.5 ± 4.9^1,2,3^	12.2 ± 6.0
Sex (M/F)	16/12	15/13	18/10	14/14	63/49
Age (year)	52.6 ± 10.5	53.2 ± 9.9	52.6 ± 9.2	49.4 ± 11.6	51.9 ± 10.4
BMI (kg/m^2^)	26.1 ± 4.3	26.7 ± 4.2	26.9 ± 3.8	27.2 ± 3.3	26.7 ± 3.9
HgbA1c (%)	8.1 ± 1.2^2,4^	7.2 ± 1.1^1,3^	8.2 ± 1.8^2,4^	6.9 ± 1.2^1,3^	7.6 ± 1.4
FPG (mmol/L)	8.6 ± 2.1	8.2 ± 2.4	9.6 ± 3.5^4^	7.2 ± 3.2^3^	8.4 ± 3.0
SBP (mmHg)	122.8 ± 13.8	127.4 ± 13.8	125.7 ± 9.5	124.6 ± 1.6	125.1 ± 14.3
DBP (mmHg)	74.4 ± 10.2	77.6 ± 12.0	78.4 ± 8.5	78.5 ± 10.5	77.2 ± 10.4
TG (mmol/L)	1.60 ± 0.89	1.58 ± 0.91	1.95 ± 0.90	1.77 ± 1.12	1.72 ± 0.96
HDL (mmol/L)	1.07 ± 0.33	1.16 ± 0.35	1.16 ± 0.62	1.05 ± 0.37	1.11 ± 0.42
LDL (mmol/L)	2.59 ± 0.93^4^	2.64 ± 0.65^4^	2.79 ± 0.86	3.14 ± 0.89^1,2^	2.79 ± 0.86
HOMA-β	32.8 ± 43.8	56.6 ± 61.5	36.6 ± 42.7	108.3 ± 79.8^1,2,3^	58.6 ± 65.5
HOMA-IR	4.76 ± 3.23	6.56 ± 5.74	7.59 ± 5.73	6.30 ± 6.37	6.25 ± 5.42
FFA (μmol/L)	83.4 ± 29.3	84.1 ± 29.1	89.6 ± 29.4	84.6 ± 24.5	85.4 ± 27.8
C-peptide (pmol/L)	676.5 ± 337.2	643.6 ± 330.5	652.1 ± 322.8	826.8 ± 378.0	700.4 ± 346.5
Adiponectin	7.70 ± 4.14	9.19 ± 7.78	8.28 ± 5.91	6.87 ± 3.63	8.01 ± 5.61

Mean ± SD; uPA: urokinase plasminogen activator; BMI: body mass index; HgbA1c: glycated hemoglobin A1c; FPG: fasting plasma glucose; SBP: systemic blood pressure; DBP: diastolic blood pressure; TG: triglyceride; HDL: high-density lipoprotein; LDL: low-density lipoprotein. ^1^ *p* < 0.05 against uPA1, ^2^ *p* < 0.05 against uPA2, ^3^ *p* < 0.05 against uPA3, ^4^ *p* < 0.05 against uPA.

## Data Availability

The datasets used and analyzed during the current study are available from the corresponding authors upon reasonable request.

## Abbreviations

AUC: area under curve; DX: X day after successful development of diabetes; ELISA: enzyme-linked immunosorbent assay; h: hour; H&E: hematoxylin and eosin; HOMA-β: homeostasis model assessment-β; HOMA-IR: homeostasis model assessment–insulin resistance; IHC: immunohistochemical; MTT: 3-(4,5-cimethylthiazol-2-yl)-2,5-diphenyl tetrazolium bromide; NTM: nicotinamide; OGTT: oral glucose-tolerance test; PCNA: proliferating cell nuclear antigen; S-siRNA: scramble siRNA; STZ: streptozotocin; T2DM: type 2 diabetes mellitus; uPA: urokinase-type plasminogen activator; uPA-/-: uPA knockout;
